# The Street-Car and the Public Health

**Published:** 1889-08

**Authors:** 


### *THE STREET-CAR AND THE PUBLIC HEALTH.*

WHEN we called attention to this important subject in the columns of the JOURNAL last March, we were confident the Board of Health would take some action looking to the improvement of the street-car service in this city. Failing to observe any such action up to the present time, we feel it a duty to again invite that body, which is the conservator of public health both as to prevention and cure, to this important and even burning question. We see no good reason why this Board should not require a rigid inspection of the street-cars, to the end that they be properly ventilated and warmed in Winter, and otherwise kept in a proper sanitary condition. Thousands of people daily ride in these cars, which, in their present condition, are, no doubt, a source of infection and contagion-carrying during the cold season, thus becoming a constant menace to public health, as well as a source of individual suffering.

One great evil is the musty hay used in Winter to deceive the passengers into the belief that their feet are kept warm thereby,—an *ignis fatuus* that dazzles to betray into the mire of pedalic refrigeration, besides serving as a source of filthiness that is disgusting to contemplate.

The *Northwestern Medical Journal*, in a late number, has discussed the relations of the street-car to the public health so ably that we feel warranted in reproducing the editorial entire:

THE STREET-CAR AND THE PUBLIC HEALTH.

No question is of greater importance to the public health of great cities than the proper construction and interior arrangement of street-cars, for they have come to take the place in our modern civilization largely of all other modes of conveyance in our great metropolitan centers of population, because of cheapness and adaptation to the wants of the traveling public, and yet with all their excellencies, there are many defects in arrangement and management very detrimental to the health of the patrons.

Not sufficient caution is taken by the officials to prevent persons from traveling who have infectious diseases, and very often persons with small-pox, measles or other contagious diseases, are found mingling with others in a crowded car, thus endangering the public health.

We know several instances of this kind, in this city, of persons on the car giving every evidence of disease which could be conveyed by such contact as could not be prevented in a crowded street-car.

If a person boards a car bearing the first evidence of contagious disease, it should be the first duty of the conductor to find out the truth in the matter at once. A notice posted in the car warning such persons against riding would be in place.

The company should make and enforce strict rules in a matter so closely related to the public welfare.

Too great caution cannot be exercised on the part of street-car officials.

Another menace to the public health is the crowded condition of the cars.

Too few cars are run to accommodate the public, and often age and youth, strong men and invalids, are packed in a small, tight car, for a long time.

Many, unable to stand, are obliged to do so, and health, and sometimes life, are both endangered, and the management, after having once secured their franchise, seem quite indifferent to the complaints of the people.

If a company are[1] able to build, equip and run a paying street-car line, they ought to be compelled by the municipality to put on cars enough to comfortably seat the traveling public, or exact no fare from those obliged to stand. Men who have stood all day at their work, ought not to peril health by being obliged to stand all the way to their homes.

It amounts almost to an insult to the public to exact fare when no seat is provided by the company. Again, too little care is exercised on the part of conductors in looking after the comforts of the patrons. Often in cold weather sickly persons are exposed to unnecessary drafts of air, and lay the foundation of incurable disease.

If cities like Minneapolis give a street railway exclusive franchise to operate, they ought, also, to compel attention to the comfort and safety of the public, from the management.

It would be well for city boards of health to give more attention to the close relation of city railway equipment and management and the public health. The same arguments will hold, also, when applied to steam railway cars and management in most particulars, although greater improvements are made for physical comfort and health on the part of steam railway management. We hope the time approaches when all the defects alluded to will be corrected.

This intelligent presentation of the subject applies with equally cogent force to the street-cars in this city, and even much more might be added without probing our case to the bottom.

1. So in original.

We return to the subject thus early, that ample time may be afforded the Board of Health to put the necessary machinery in operation to correct the evils pertaining to this great public calamity before cold weather sets in, and much irreparable damage done.

We hope before new franchises are granted to any company, the city authorities will take such action as will protect itself against a repetition of the dangerous conditions complained of, and which are allowed to continue by the present monopoly without heed.

